# Anal Cancer Incidence Among Women With a History of Cervical Cancer by Age and Time Since Diagnosis

**DOI:** 10.1001/jamanetworkopen.2025.31362

**Published:** 2025-09-11

**Authors:** Haluk Damgacioglu, Chandler Curtis, Kalyani Sonawane, Gary Clifford, Joel M. Palefsky, Elizabeth Y. Chiao, Keith Sigel, Ashish A. Deshmukh

**Affiliations:** 1 Department of Public Health Sciences, Medical University of South Carolina, Charleston; 2 Cancer Control Program, Hollings Cancer Center, Medical University of South Carolina, Charleston; 3 Early Detection, Prevention and Infections, International Agency of Research on Cancer, Lyon, France; 4 Department of Medicine, University of California, San Francisco, San Francisco; 5 Department of Epidemiology, MD Anderson Cancer Center, Houston, Texas; 6 Department of General Internal Medicine, Mount Sinai Icahn School of Medicine, New York, New York

## Abstract

This cohort study estimates anal cancer incidence in women with a history of cervical cancer, by age and time since diagnosis.

## Introduction

Women with a history of cervical cancer have a higher anal cancer incidence (9 vs 2 cases per 100 000) than the general population.^1,2^ While anal cancer screening is recommended for other high-risk groups—people with HIV (after age 35 years), women with a history of vulvar cancer (within 1 year of diagnosis), and solid organ transplant recipients (10 years posttransplant)—guidance for women with a history of cervical cancer is limited due a lack of age-based or duration-based data. We estimated anal cancer incidence in this population by age and time since cervical cancer diagnosis.

## Methods

In this cohort study, we followed women with cervical cancer using data from the National Cancer Institute’s Surveillance, Epidemiology, and End Results (SEER)–8 registries^3^ (1975-1999) and SEER-17 registries^4^ (2000-2021). Cervical cancer cases were identified using the *International Classification of Diseases for Oncology, 3rd edition* sites C53.0, C53.1, C53.8, and C53.9, as well as histology codes 8010 to 8617 and 8940 to 8941. Among these women, anal cancer diagnoses were identified using sites C21.0 to C21.2, C21.8, and histology codes 8050 to 8084 and 8120 to 8131. To define a cohort at risk for incident anal cancer following cervical cancer, women diagnosed with anal cancer before or within 2 months of cervical cancer diagnosis were excluded. Overall, age-specific, and time since diagnosis–specific incidence rates (IRs) were estimated using the multiple primary standardized incidence ratios session in SEER*Stat version 8.4.5 software, with rates reported per 100 000 person-years. Standardized incidence ratios, adjusted for race and/or ethnicity, age, and calendar year, were used to compare anal cancer risk with the general population. This study followed STROBE reporting guideline and was deemed exempt from review and the need for informed consent by the Medical University of South Carolina institutional review board, as it used deidentified, publicly available data, in accordance with 45 CFR §46. Analyses were conducted between February and June 2025.

## Results

A total of 85 524 women diagnosed with cervical cancer between 1975 and 2021 were identified, with a follow-up of 822 630 person-years. Among these women, 64 cases of anal cancer were diagnosed. Overall, anal cancer incidence was 7.8 cases per 100 000 person-years (95% CI,  6.1-9.9 cases per 100 000 person-years), with differences across age groups and time since cervical cancer diagnosis (Table). Among women younger than 45 years with a history of cervical cancer, the IR was 2.4 (95% CI,  1.0-5.7). This rate increased to 4.6 (95% CI,  2.5-8.5) for women aged 45 to 54 years and 10.0 (95% CI,  6.4-15.8) for those aged 55 to 64 years. The highest IR was observed in women aged 65 to 74 years, at 17.6 (95% CI,  11.6-26.7), while the IR for women aged 75 years or older was 10.0 (95% CI,  5.0-20.0) (Figure).

**Table.** Overall and Age-Specific Anal Cancer Incidence and Relative Risk Among Women With a History of Cervical Cancer

Variable	Anal cancer cases, No.	Person-years, No.	Incidence rate, cases/100 000 person-years (95% CI)	SIR (95% CI)
Overall	64	822 630	7.8 (6.1-9.9)	1.9 (1.5-2.5)
Age at anal cancer diagnosis, y				
<45	5	80 098	2.4 (1.0-5.7)	3.5 (1.1-8.1)
45-54	10	217 783	4.6 (2.5-8.5)	1.5 (0.7-2.8)
55-64	19	189 122	10.0 (6.4-15.8)	1.8 (1.1-2.8)
65-74	22	125 127	17.6 (11.6-26.7)	2.5 (1.6-3.8)
≥75	8	80 098	10.0 (5.0-20.0)	1.5 (0.6-2.9)
Time since cervical cancer diagnosis, y				
<5	17	292 094	5.8 (3.6-9.4)	2.0 (1.2-3.2)
5 to <10	10	196 078	5.1 (2.7-9.5)	1.4 (0.7-2.7)
10 to <15	8	136 106	5.9 (2.9-11.8	1.4 (0.6-2.7)
15 to <20	11	86 536	12.7 (7.0-23.0)	2.5 (1.3-4.5)
≥20	18	111 816	16.1 (10.1-25.6)	2.4 (1.4-3.9)

Abbreviation: SIR, standardized incidence ratio.

**Figure.**
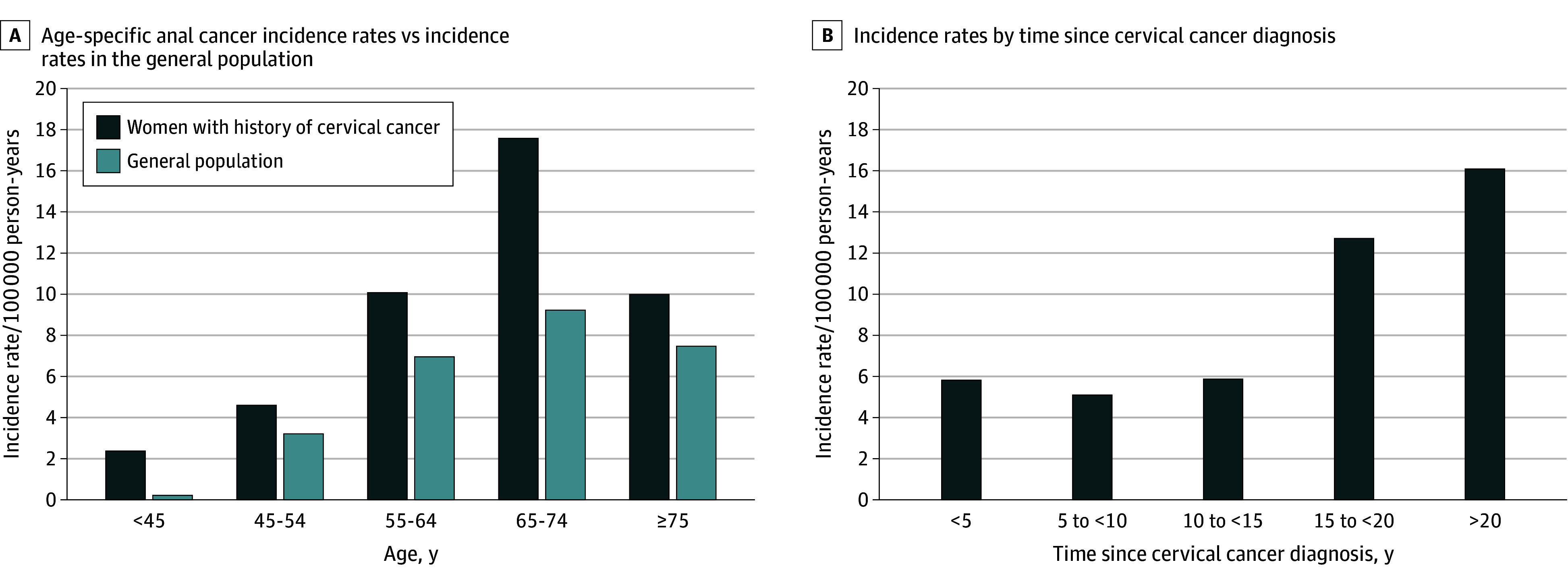
Anal Cancer Incidence Among Women With a History of Cervical Cancer A, Age-specific incidence rates compared with incidence rates in the general population. The anal cancer incidence for women in the general population in 2021 was used for comparison. B, Incidence rates by time since cervical cancer diagnosis.

Anal cancer incidence varied with time since cervical cancer diagnosis. For women diagnosed with cervical cancer, the incidence in the first 5 years was 5.8 (95% CI,  3.6-9.4). IRs for the time since cervical cancer diagnosis were 5.1 (95% CI,  2.7-9.5) for 5 to 10 years, 12.7 (95% CI,  7.0-23.0) for 10 to 15 years, and 16.1 (95% CI,  10.1-25.6) for greater than or equal to 20 years (Figure). Fifty-nine percent of anal cancer cases diagnosed among women aged 65 to 74 occurred more than 15 years after their cervical cancer diagnosis. The overall standardized incidence ratio for anal cancer was 1.9 (95% CI,  1.5-2.5). Elevated risk persisted over time, with notably high standardized incidence ratios observed 15 to 20 years (2.5; 95% CI,  1.3-4.5) and 20 years or more (2.4; 95% CI,  1.4-3.9) after cervical cancer diagnosis.

## Discussion

This cohort study suggests that anal cancer incidence exceeds the rate of 17 cases per 100 000—a critical threshold identified for recommending screening use—among women aged 65 to 74 years with a history of cervical cancer.^5^ Additionally, incidence increases with increasing duration from the index cervical cancer, reaching 16.1 cases per 100 000 among women after 20 years from the index cervical cancer diagnosis. These findings support the need for age-based and duration-based anal cancer screening recommendations for women with cervical cancer history. While this study provides objective data to define risk for screening eligibility, future research is important to understand screening benefits and harms and determine the optimal age to initiate screening and optimal screening intervals. A limitation is the lack of data on risk factors (eg, human papillomavirus infection) to help explain the association between cervical and anal cancers. In summary, this study provides critical data to inform anal cancer screening recommendations among a high-risk population of women with a history of cervical cancer.

## Notes

Supplement.
Data Sharing Statement

## References

[1] Deshmukh AA, Suk R, Shiels MS, . Recent trends in squamous cell carcinoma of the anus incidence and mortality in the United States, 2001–2015. J Natl Cancer Inst. 2020;112(8):829-838. doi:10.1093/jnci/djz219 31742639 PMC7825484

[2] Clifford GM, Georges D, Shiels MS, . A meta-analysis of anal cancer incidence by risk group: toward a unified anal cancer risk scale. Int J Cancer. 2021;148(1):38-47. doi:10.1002/ijc.33185 32621759 PMC7689909

[3] National Cancer Institute: Surveillance, Epidemiology, and End Results Program. SEER*stat database: incidence - SEER research data, 8 registries, nov 2023 sub (1975-2021. 2024. Accessed July 25, 2025. https://seer.cancer.gov/data-software/documentation/seerstat/nov2023/

[4] National Cancer Institute: Surveillance, Epidemiology, and End Results Program. SEER*stat database: incidence - SEER research data, 17 registries (excluding AK), nov 2023 sub (2000-2021) - linked to county attributes - total U.S., 1969-2022 counties. 2024. Accessed July 25, 2025. https://seer.cancer.gov/data-software/documentation/seerstat/nov2023/

[5] Stier EA, Clarke MA, Deshmukh AA, . International Anal Neoplasia Society’s consensus guidelines for anal cancer screening. Int J Cancer. 2024;154(10):1694-1702. doi:10.1002/ijc.34850 38297406

