# Supplementary material for: Anal Cancer Incidence Among Women With a History of Cervical Cancer by Age and Time Since Diagnosis
Source: JAMA Netw Open. 2025 Sep 11;8(9):e2531362. doi: 10.1001/jamanetworkopen.2025.31362 (PMC12426789; doi:10.1001/jamanetworkopen.2025.31362)
Supplement: Supplement. — Data Sharing Statement [file jamanetwopen-e2531362-s001.pdf]

Data Sharing Statement

Damgacioglu. Anal Cancer Incidence Among Women With a History of Cervical Cancer by Age and Time Since Diagnosis. *JAMA Netw Open*. Published September 11, 2025. doi:10.1001/jamanetworkopen.2025.31362

Data
Data available: No
